# Increased angiogenesis parallels cardiac tissue remodelling in experimental acute *Trypanosoma cruzi* infection

**DOI:** 10.1590/0074-02760220005

**Published:** 2022-11-21

**Authors:** Lindice Mitie Nisimura, Roberto Rodrigues Ferreira, Laura Lacerda Coelho, Elen Mello de Souza, Beatriz Matheus Gonzaga, Patrícia Mello Ferrão, Mariana Caldas Waghabi, Liliane Batista de Mesquita, Mirian Claudia de Souza Pereira, Otacilio da Cruz Moreira, Joseli Lannes-Vieira, Luciana Ribeiro Garzoni

**Affiliations:** 1 Fundação Oswaldo Cruz-Fiocruz, Instituto Oswaldo Cruz, Laboratório de Inovações em Terapias, Ensino e Bioprodutos, Rio de Janeiro, RJ, Brasil; 2 Fundação Oswaldo Cruz-Fiocruz, Instituto Oswaldo Cruz, Laboratório de Genômica Funcional e Bioinformática, Rio de Janeiro, RJ, Brasil; 3 Fundação Oswaldo Cruz-Fiocruz, Instituto Oswaldo Cruz, Laboratório de Virologia Molecular, Rio de Janeiro, RJ, Brasil; 4 Fundação Oswaldo Cruz-Fiocruz, Instituto Oswaldo Cruz, Laboratório de Ultraestrutura Celular, Rio de Janeiro, RJ, Brasil; 5 Fundação Oswaldo Cruz-Fiocruz, Instituto Oswaldo Cruz, Laboratório de Biologia Molecular e Doenças Endêmicas, Rio de Janeiro, RJ, Brasil; 6 Fundação Oswaldo Cruz-Fiocruz, Instituto Oswaldo Cruz, Laboratório de Biologia das Interações, Rio de Janeiro, RJ, Brasil

**Keywords:** cardiac angiogenesis, cardiac tissue remodelling, experimental acute Chagas disease

## Abstract

**BACKGROUND:**

Angiogenesis has been implicated in tissue injury in several noninfectious diseases, but its role in Chagas disease (CD) physiopathology is unclear.

**OBJECTIVES:**

The present study aimed to investigate the effect of *Trypanosoma cruzi* infection on cardiac angiogenesis during the acute phase of experimental CD.

**METHODS:**

The signalling pathway involved in blood vessel formation and cardiac remodelling was evaluated in Swiss Webster mice infected with the Y strain of *T. cruzi*. The levels of molecules involved in the regulation of angiogenesis, such as vascular endothelial growth factor-A (VEGF-A), Flk-1, phosphorylated extracellular-signal-regulated protein kinase (pERK), hypoxia-inducible factor-1α (HIF-1α), CD31, α-smooth muscle actin (α-SMA) and also the blood vessel growth were analysed during *T. cruzi* infection. Hearts were analysed using conventional histopathology, immunohistochemistry and western blotting.

**FINDINGS:**

In this study, our data demonstrate that *T. cruzi* acute infection in mice induces exacerbated angiogenesis in the heart and parallels cardiac remodelling. In comparison with noninfected controls, the cardiac tissue of *T. cruzi*-infected mice presented higher levels of (i) HIF-1α, VEGF-A, Flk-1 and pERK; (ii) angiogenesis; (iii) α-SMA^+^ cells in the tissue; and (iv) collagen -1 deposition around blood vessels and infiltrating throughout the myocardium.

**MAIN CONCLUSIONS:**

We observed cardiac angiogenesis during acute experimental *T. cruzi* infection parallels cardiac inflammation and remodelling.

Chagas disease (CD), caused by the protozoan *Trypanosoma cruzi*, is neglected and affects about six to seven million people worldwide.^1^ Chronic cardiomyopathy is the main clinical manifestation of CD. Myocardial remodelling occurs as a consequence of progressive cellular death, initiated and perpetuated by early alterations in heart microcirculation.^2^

Microvascular alterations are involved in the pathogenesis of CD, both in experimental models and in humans. ^(^ ^2^ ^-^ ^5^ Recently, we reported cerebral microvasculopathy in mice during experimental acute CD. *T. cruzi* infected animals presented cerebral microvascular dysfunction, obstructive plugging, microvascular inflammation and functional capillary rarefaction.^4^ In the heart of patients with CD, vascular constrictions, microaneurysms, dilatation and occlusive thrombi are consequences of vasoactive substances such as endothelin-1 and thromboxane.^6^ ^-^ ^8^ Moreover, cardiac angiogenesis has been described in both experimental *T. cruzi* infection in mice^9^ ^)^ and chronic Chagas cardiomyopathy in humans.^10^

Angiogenesis is a multistep process involving endothelial cell activation, vascular instability caused by detaching of mural cells (smooth muscle cells), cell proliferation, extracellular matrix (ECM) degradation, endothelial cell migration, tube formation and vascular maturation by recruitment of mural cells. Changes in the physiological regulation of angiogenesis can result in disorders including inflammation, ischemia, and tumour growth. ^(^ ^11^ One of the key mediators of angiogenesis is the vascular endothelial growth factor-A (VEGF-A), which is induced by hypoxia-inducible factor-1α (HIF-1α). VEGF-A activates receptor tyrosine kinase VEGFR-2/Flk-1, inducing extracellular-signal-regulated protein kinase (ERK) 1/2 phosphorylation, cellular proliferation and vascular growth.^11^ VEGF-A is also a potent inducer of vascular permeability.^12^ Finally, vascular maturation is achieved by the recruitment of pericytes or smooth muscle cells which express α-smooth muscle actin (α-SMA).^13^ ^,^ ^14^

Both tissue ischemia and inflammation contribute to vascular growth. However, contrary to what is observed in ischemic diseases, angiogenesis in chronic inflammatory disorders is exacerbated and abnormal, contributing to pathogenesis since alteration in recruitment and attachment of mural cells to the endothelium can generate and maintain chronic fibrotic processes.^15^ ^,^ ^16^

Therefore, we investigated the effect of *T. cruzi* infection on cardiac angiogenesis during the acute phase of experimental CD in mice, studying the presence of blood vessels and the canonical molecular circuit involved in neoangiogenesis.

## MATERIALS AND METHODS

*Animals -* Outbred male Swiss Webster mice (18-20g) were obtained from Fundação Oswaldo Cruz (Fiocruz) animal facilities [Centro de Criação de Animais de Laboratórios (CECAL), Rio de Janeiro, Brazil)]. The use of animals and experimental procedures is in accordance with Brazilian Law 11.794/2008 and regulations of Conselho Nacional de Controle de Experimentação Animal (CONCEA). The mice were housed at a maximum of five individuals per cage, kept in a specific-pathogen-free (SPF) room at 20 to 24°C under a 12- light and 12-h dark cycle and provided sterilised water and chow *ad libitum*. All animal experimental procedures were performed following the license (LW - 40/13) approved by the ethics committee for animal use at Comissão de Ética no Uso de Animais (CEUA/Fiocruz) and were consistent with the U.S. National Institutes of Health Guide for the Care and Use of Laboratory Animals (National Research Council Committee for the Update of the Guide for the and Use of Laboratory, 2011) and accordance of the Ethics - European Commission (Directive 2010/63/EU). Mice were inoculated intraperitoneally with 10^4^ *T. cruzi* bloodstream trypomastigotes forms (Y strain) and divided in two groups: noninfected (n = 5) and *T. cruzi* infected (n = 15-20). On the respective day post-experimental infection with *T. cruzi,* all animals were euthanised. To preserve animal welfare (due to parasite infection), daily observation was supervised by a Ph.D. veterinarian aiming to avoid animal suffering and pain. All experiments followed humane endpoints through euthanasia that presented suffering such as disturbed motor and exploratory activity and/or moribund condition. Three independent experiments were performed. 

*Parasitological parameters -* Blood examination was followed daily by light microscopy for parasitemia evaluation, as previously described.^17^ Mortality was monitored regularly for 22 days post-infection (dpi). 

*Histopathology -* Animal hearts were obtained at 8, 15 and 22 dpi (five animals per day), then included in paraffin for evaluation of tissue parasitism, inﬂammatory inﬁltrates and blood vessel number. Hearts were sectioned (5 µm thick) in microtome, stained with haematoxylin/eosin or Masson’s trichrome and examined by light microscopy. The vessel quantification by light microscopy or conventional fluorescence microscopy was performed by the morphology aspect of the vessel in at least 10 fields per slice from five animals for group.

*Immunofluorescence -* Paraffin-embedded or optimal cutting temperature compound (OCT compound) frozen heart sections (three-five animals/group) from control or infected (15th dpi) mice were stained with specific primary antibodies [rabbit anti-VEGF-A (sc-507) and anti-PECAM-1 (sc-46694) from Santa Cruz, Dallas, USA; mouse anti-α-SMA (A2547), from Sigma, San Luis, USA] and secondary antibodies [goat anti-mouse (A-21202) and goat anti-rabbit (A-21206) Alexa Fluor 488 from Thermo Fisher Scientific, Waltham, USA]. DNA was stained with 4’,6-diamidino-2-phenylindole (DAPI). Slides were examined in Zeiss Axioplan 2 microscope equipped with epiﬂuorescence and LSM 510 Zeiss confocal microscope and analysis was executed in the reconstructed images from five animals for group. The software Image J was used for fluorescence intensity quantification analysis. Further image processing was performed with Adobe Photoshop software (Adobe Systems Inc., San Jose, USA).

*Immunoblot analysis -* Ventricular heart proteins from each group (NI and Y) were extracted from 100 mg tissue/mL phosphate-buffered saline (PBS), to which 0.4 M sodium chloride, 0.05% Tween 20 and protease inhibitors (0.1 mM phenylmethylsulfonyl fluoride and 1/100 protease and phosphatase inhibitors cocktail - Sigma) were added. The samples were sonicated twice and centrifuged for 10 min at 3,000 *g* and the supernatant was kept frozen at -80^o^C. Total proteins in the lysates (20-40 µg/lane) were separated by sodium dodecyl sulfate polyacrylamide gel electrophoresis (SDS/PAGE) (10 or 12%) and transferred to nitrocellulose membranes (Hybond C, GE Healthcare, Chicago, USA). Nonspecific binding sites were blocked by incubating the membranes with 5% (w/v) nonfat milk/tris buffered saline (TBS)/0.1% Tween-20 for 1 h at room temperature. The membranes were probed with specific primary antibodies rabbit anti-VEGF-A (sc-507) (42 kDa), rabbit anti-HIF-1α (sc -12542) (132 kDa), mouse anti-Flk-1(sc-6251) (150 kDa) and rabbit anti-pERK1/2 (sc-101760) (42 and 44 kDa), all from Santa Cruz. For loading control, mouse anti-Glyceraldehyde 3-phosphate dehydrogenase (GAPDH) (36 kDa) monoclonal antibody from Fitzgerald, Concord, USA (10R-109a) was used. The membranes were incubated with secondary goat anti-rabbit IgG (31460) or goat anti-mouse IgG (31430) horseradish peroxidase (HRP)-labelled antibody for 1-2 h at 25°C, followed by incubation with chemiluminescent SuperSignal kit (Thermo Fisher Scientific).

*Statistical analysis -* Statistical analyses were performed using the unpaired *t* test or one-way analysis of variance (ANOVA) and Tukey’s post-test with a significance level of p < 0.05. The data represent the mean ± standard error of the mean (SEM) of three independent experiments. All statistical analysis was performed using GraphPad InStat 5.0 (GraphPad Software Inc., La Jolla, USA).

*Ethics statement -* The use of animals and experimental procedures is in accordance with Brazilian Law 11.794/2008 and regulations of CONCEA. All animal experimental procedures were performed following the license (LW - 40/13) approved by CEUA/Fiocruz and were consistent with the U.S. National Institutes of Health Guide for the Care and Use of Laboratory Animals (National Research Council Committee for the Update of the Guide for the and Use of Laboratory, 2011) and accordance of the Ethics - European Commission (Directive 2010/63/EU).

## RESULTS

**Fig. 1:**
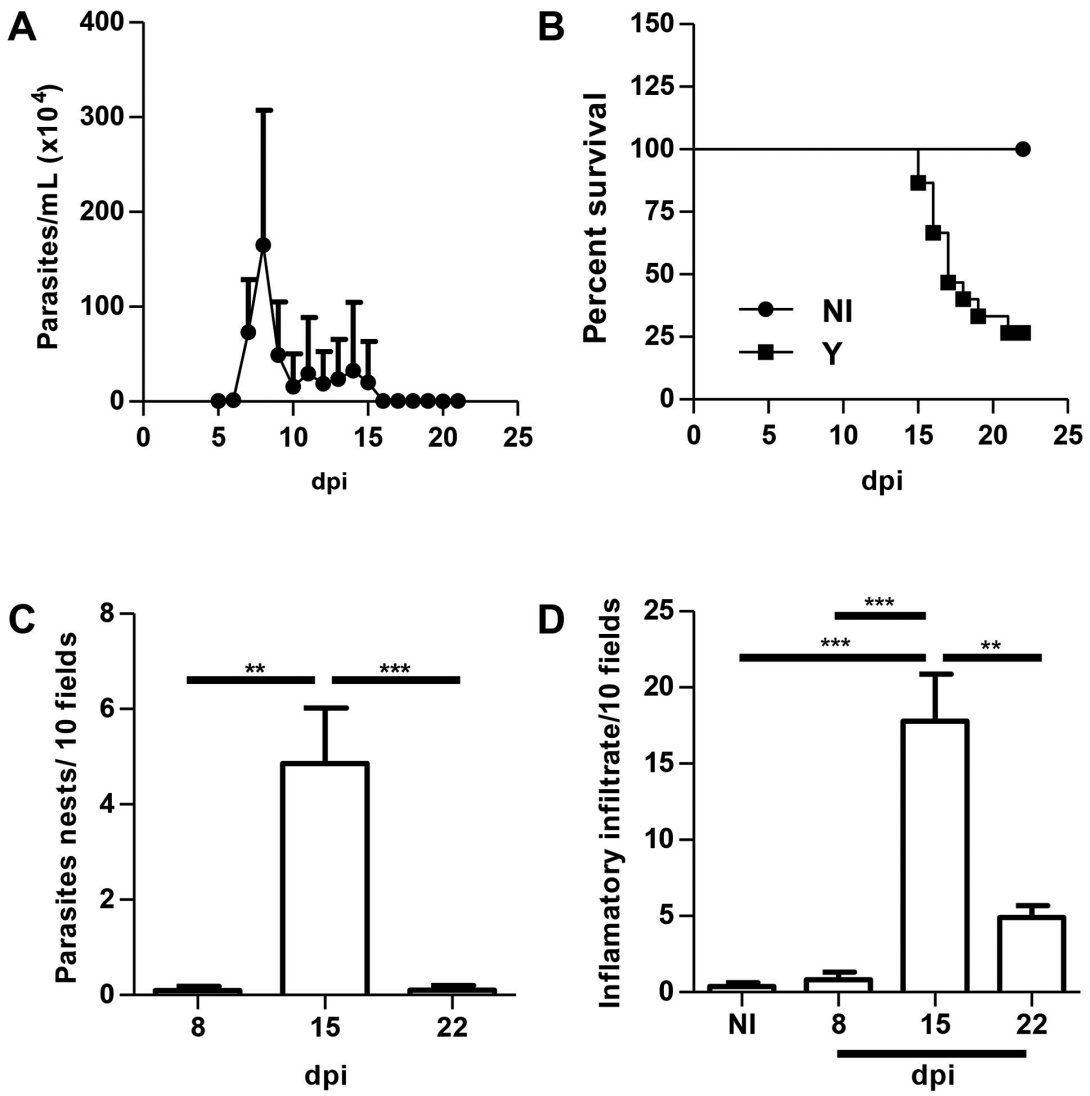
acute Chagas disease (CD) in Swiss Webster mice infected with the Y strain of *T. cruzi.* Mice were infected with 10^4^ blood trypomastigote forms and the following parameters were evaluated in a kinetic study: parasitemia (A), survival rate (B), tissue parasitism (C) and inflammatory infiltrates (D). Mean ± standard error of the mean (SEM). One-way analysis of variance (ANOVA) test, p < 0.01^**^ and p < 0.001^***^. N = 20. dpi: days post-infection; NI: noninfected.

*Experimental acute CD in mice -* Evaluation of parasitemia revealed that the peak of *T. cruzi* trypomastigote forms occurred at 8 dpi (Fig. 1A). Only 20% of *T. cruzi* infected animals survived at 22 dpi (Fig. 1B). We also evaluated the cardiac parasitism and inflammation at 8, 15 and 22 dpi. The peak of tissue parasitism and inflammation occurred at 15 dpi (Fig. 1C and 1D). A significant reduction of both amastigote nests and inflammatory infiltrates was observed in the myocardium of infected mice at 22 dpi (Fig. 1C and 1D). 

**Fig. 2:**
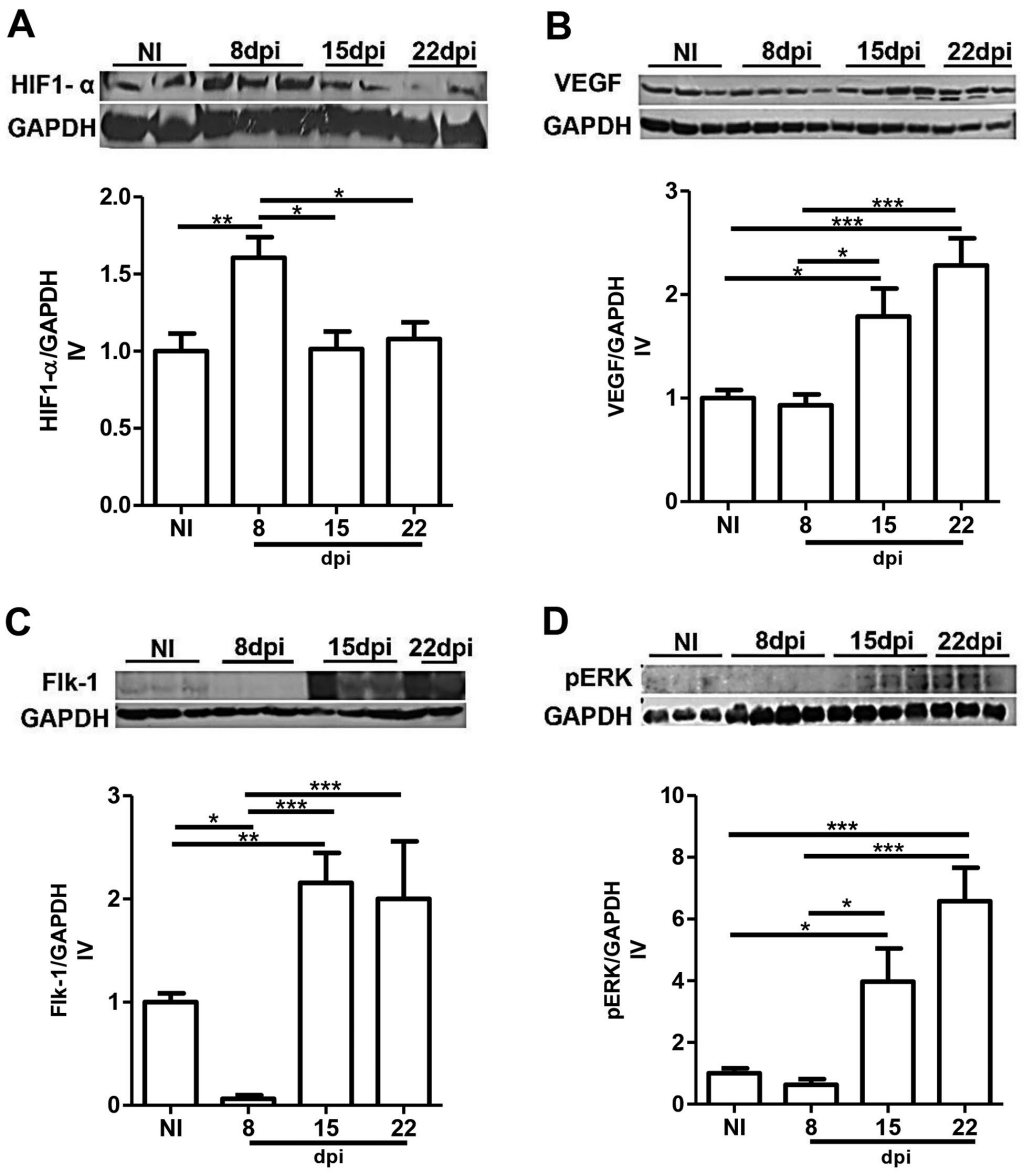
western blotting of proangiogenic proteins in the heart. Representative immunoblot analysis of hypoxia-inducible factor-1α (HIF-1α) (132 kDa) (A), vascular endothelial growth factor (VEGF) (42 kDa) (B), Flk-1 (150 kDa) (C) and phosphorylated extracellular-signal-regulated protein kinase (pERK) 1/2 (44 kDa) (D) levels in cardiac tissue. Hearts of noninfected (NI) mice and *Trypanosoma cruzi* infected at 8, 15 and 22 days post-infection (dpi) were harvested, and protein lysates were probed with anti-HIF-1α, anti-VEGF, anti-Flk-1 and anti-pERK1/2 antibodies. Glyceraldehyde 3-phosphate dehydrogenase (GAPDH) (36 kDa) was used as a loading control. One-way analysis of variance (ANOVA) test, p < 0.05^*^, p < 0.01** and p < 0.001^***^. N = 5 for each group. IV: index of variation of the mean ± standard error of the mean (SEM).

*Cardiac angiogenic protein levels during T. cruzi infection -* Since it has been demonstrated that HIF-1α, a marker of hypoxia, induces angiogenesis,^18^ we investigated the levels of this protein during *T. cruzi* infection by immunoblotting assay. Differential levels of HIF-1α were noticed during the course of infection in mice. Up-regulation of HIF-1α, achieving a 0.6-fold increase in the protein level, was observed in the myocardium of *T. cruzi*-infected mice at 8 dpi when compared to noninfected controls (p < 0.01). Curiously, HIF-1α levels were similar to noninfected animals at later stages of infection. We observed, at 15 and 22 dpi, differences of 0.4-fold (p < 0.05) and 0.3-fold (p < 0.05), respectively, when compared to 8 dpi (Fig. 2A). 

Next, we determined the expression of VEGF-A, a key protagonist of angiogenic processes, in the cardiac tissue by immunoblot analysis since the HIF-1α was increased. Classically, HIF-1α induces genes up-regulation, including VEGF-A.^19^ However, the higher HIF-1α observed at 8 dpi was not followed by higher levels of VEGF-A at the same time point. Our results revealed no changes in VEGF-A expression at 8 dpi compared to noninfected mice. Furthermore, contrasting to nonaltered HIF-1α when compared to the noninfected controls, the VEGF expression was up-regulated in the heart tissue of *T. cruzi*-infected mice at 15 and 22 dpi, reaching approximately 0.8-fold (p < 0.05) and 1.3-folds (p < 0.001), respectively. These levels are even higher when compared to 8 dpi, showing an increase of 0.9-fold (p < 0.05) and 1.4-folds (p < 0.001) at 15 and 22 dpi, respectively (Fig. 2B).

We also examined the impact of *T. cruzi* infection on VEGF receptor (Flk-1) levels. Our results showed a lower quantity of Flk-1 at 8 dpi (0.9-fold) and higher levels at 15 dpi (1.1-fold) (p < 0.001), when compared to the noninfected group, which was maintained at 22 dpi (Fig. 2C). Considering that both VEGF levels and its receptor were positively modulated, we evaluated the VEGF signalling pathway at a later time of infection. Then, levels of pERK 1/2 were analysed in healthy and *T. cruzi*-infected mice. *T. cruzi* infection induced ERK1/2 activation that was noticed by up-regulation of pERK with a significantly higher level of approximately 3-folds (p < 0.05) and 5.6-folds (p < 0.001) at 15 and 22 dpi when compared to noninfected mice (Fig. 2D). 

**Fig. 3:**
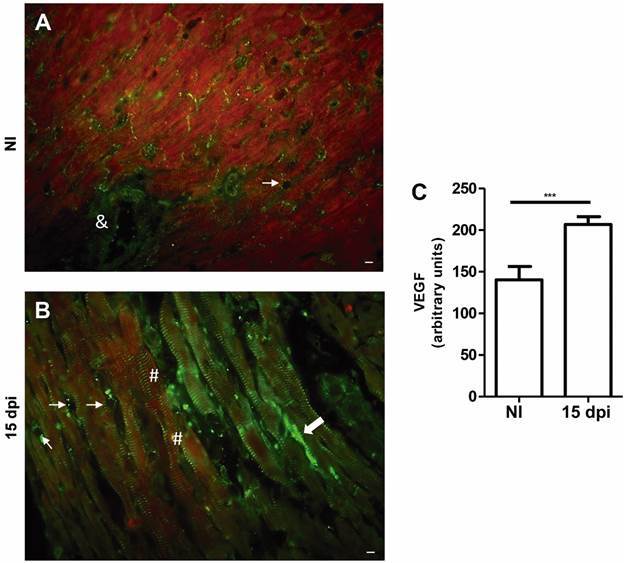
immunofluorescence of vascular endothelial growth factor (VEGF) in cardiac tissue. VEGF distribution (green) in noninfected (NI) (A) and *Trypanosoma cruzi* infected (B). NI mice displaying VEGF in blood vessels among cardiac fibres (thin arrows) and arterioles (&) (A). In *T. cruzi* infected tissues, VEGF can be observed in blood vessels (thin arrows), in areas of connective tissue (thick arrows) and diffuse in the cytoplasm of cardiomyocytes (B). Notice the VEGF striated staining pattern in myofibrils (#). Bar graph shows the quantification of fluorescence intensity (C). Mean ± standard error of the mean (SEM). Unpaired *t* test, p < 0.001^***^. Bars = 20 µm. N = 3 for each group. dpi: days post-infection.

*Immunofluorescence of VEGF in the cardiac tissue during T. cruzi infection -* The distribution of VEGF at 15 dpi was also assessed in heart tissue. Uninfected mice displayed VEGF labelling in blood vessels, between cardiac fibres and in cardiomyocytes (Fig. 3A). A more expressive VEGF labelling was detected in the heart tissue of infected animals (Fig. 3B). The quantification of fluorescence intensity revealed a 0.5-fold increase in VEGF levels (p < 0.001) in *T. cruzi* infected animal hearts (Fig. 3C).

**Fig. 4:**
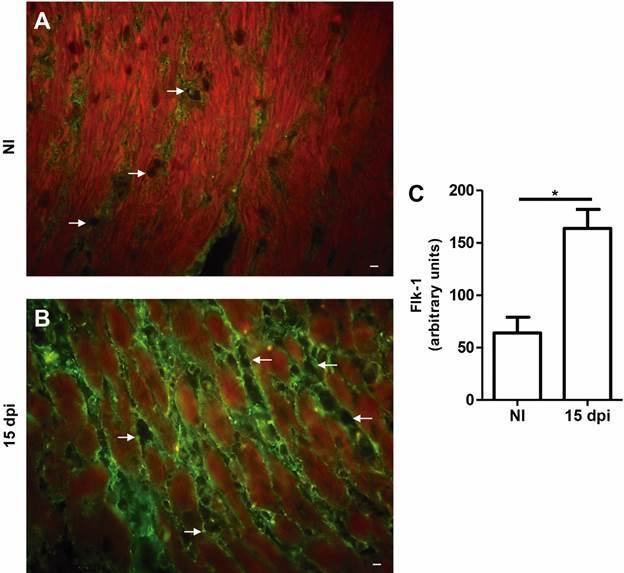
immunohistochemistry of cardiac Flk-1. Flk-1 content (green) in noninfected (NI) (A) and *Trypanosoma cruzi* infected (B) heart. NI mice heart reveals weak labelling for Flk-1 in the blood vessel (thin arrows) located between cardiac fibres (A), whereas 15 days post-infection (dpi) infected animals exhibit intense Flk-1 staining in the blood vessel (thin arrows) (B). Bar graph shows the percentage of stained area quantification (C). Mean ± standard error of the mean (SEM). One-way analysis of variance (ANOVA) test, p < 0.05*. Bars = 20 µm. N = 3 for each group.

*Immunohistochemistry of Flk-1 -* Thereafter, Flk-1 content was investigated in cardiac tissue. The Flk-1 detection showed strong labelling in the heart of infected mice after 15 days of infection when compared to cardiac tissue of noninfected animals (Fig. 4A and 4B). The quantification analysis revealed a 1.7-fold increase in Flk-1 content at 15 dpi infected animals when compared to noninfected ones (p < 0.001 and p < 0.01, respectively) (Fig. 4C).

**Fig. 5:**
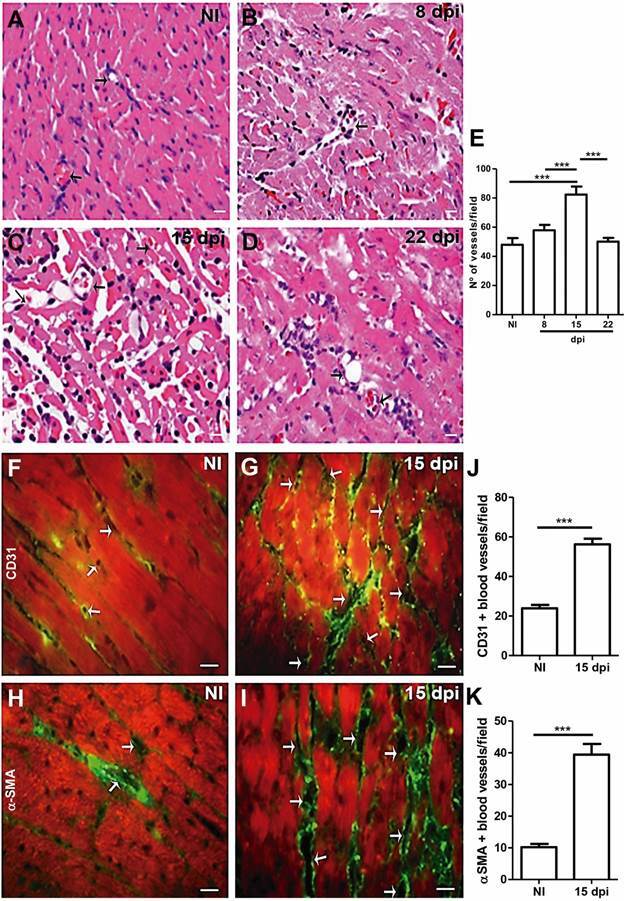
evaluation of cardiac angiogenesis. Hematoxylin and eosin (H&E) staining of noninfected (NI) (A) and *Trypanosoma cruzi* infected at 8 (B), 15 (C) and 22 days post-infection (dpi) (D), showing blood vessels (arrow). By immunofluorescence of NI (left panel) and *T. cruzi* infected cardiac tissues (right panel) (E), we can observe an increase in CD31 (F, G and J) and α-smooth muscle actin (α-SMA) positive blood vessel (arrow) in infected tissue (H, I and K), indicating angiogenesis and vascular maturation. Mean ± standard error of the mean (SEM). Unpaired *t* test, p < 0.001^***^. N = 5 for each group. Bar = 10 µm.

*Angiogenesis and collagen deposition in the heart tissue -* We further investigated the number of blood vessels in the heart under normal and pathological conditions. Heart tissue stained with hematoxylin and eosin (H&E) demonstrated that the number of blood vessels remained unaltered at 8 dpi while a significant increase of 0.7-fold was evidenced at 15 dpi compared to age-matched controls. The peak in the total number of blood vessels at 15 dpi reduced 0.4-fold at 22 dpi, returning to levels similar to control (Fig. 5E). Since CD31 [platelet endothelial cell adhesion molecule (PECAM)] has been utilized as an angiogenesis marker, we also performed immunohistochemical analysis to evaluate blood vessel distribution in the cardiac tissue of both healthy and *T. cruzi* infected mice (Fig. 5F and 5G). Our analysis revealed an elevated number of CD31 reactive vessels (1.3-fold the control level; p < 0.001) in the cardiac tissue of infected mice at 15 dpi (Fig. 5J), supporting the quantitative data obtained with H&E-stained tissue.

**Fig. 6:**
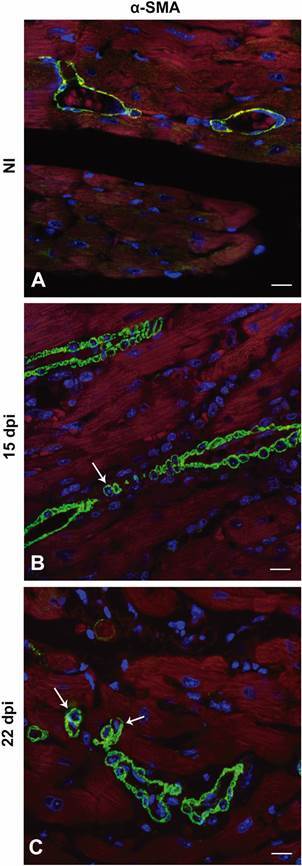
confocal microscopy analysis of α-smooth muscle actin (α-SMA) positive cells. Immunostaining (green) of α-SMA shows the mural cells in arterioles of cardiac tissue (A-C). A continuous staining pattern can be observed in the controls (A), while in infected hearts the α-SMA staining is clearly delimited around individuals myofibroblasts near blood vessels 15 and 22 days post-infection (dpi) (arrow) (B and C). Bar = 10 µm. N = 5 for each group. NI: noninfected.

**Fig. 7:**
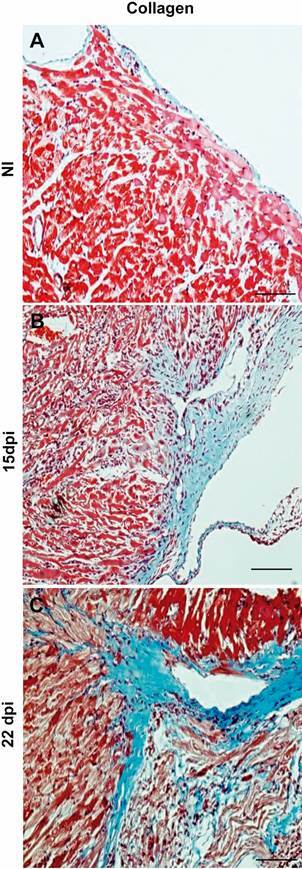
analysis of fibrosis by histochemistry. Masson’s trichrome staining shows the collagen distribution in noninfected (NI) (A) and in *T. cruzi* infected hearts at 15 (B) and 22 (C) days post-infection (dpi). Notice the strong collagen staining around blood vessels infiltrating through the cardiac tissues in infected animals. Bar = 10 µm. N = 5 for each group.

Next, we analysed the presence of α-SMA positive cells in the cardiac tissue of noninfected and *T. cruzi*-infected mice (Fig. 5H-I). Vascular maturation is the last step of the angiogenesis process. It occurs through the covering of the external vascular surface with α-SMA positive pericytes and vascular smooth muscle cells.^20^ At 15 dpi, immunohistochemical analysis revealed a higher number of α-SMA positive blood vessels in the cardiac tissue of infected mice (Fig. 5I) when compared to noninfected mice (Fig. 5H). Quantification revealed a 2.8-fold increase in infected animals (p < 0.0001) (Fig. 5K). At 15 and 22 dpi, we observed α-SMA+ cells scattering near vascular structures and infiltrating into the myocardium (Fig. 6B and 6C). Additionally, Masson’s trichrome staining of *T. cruzi*-infected myocardium (15 and 22 dpi) demonstrated an intense deposit of collagen fibres compared to cardiac tissue of noninfected mice, near blood vessels and infiltrating the cardiac tissue (Fig. 7B and 7C).

## DISCUSSION

In this study, we investigated the effect of *T. cruzi* infection on cardiac angiogenesis. Our experiments show, for the first time, that experimental acute *T. cruzi* infection alters the regulation of angiogenic proteins including HIF-1α, VEGF-A and Flk-1 in the heart tissue and induces exacerbated transient angiogenesis. We also observed, in *T. cruzi* infected animals, α-SMA+ cells (myofibroblasts) near blood vessels and spreading through cardiac tissue. It may suggest the vascular origin of these cells as described in other diseases, in which fibrosis has a vascular component. Chagas cardiomyopathy has an important inflammatory component^21^ ^-^ ^26^ and, currently, there are several indicators that angiogenesis is involved in fibrogenesis in inflammatory disorders.^15^ ^,^ ^16^

VEGF-A regulates the growth and survival of endothelial cells, playing a critical role in both physiological and pathological angiogenesis.^20^ The effects of VEGF-A occur mainly through its receptor Flk-1 and involve the activation of multiple signalling pathways, including ERK, which regulates the proliferation of endothelial cells.^20^ VEGF-A is also implicated in the increase of vascular permeability.^12^ This protein is up-regulated by hypoxia, via induction of the transcription factor HIF-1α.^19^ ^,^ ^27^ ^-^ ^30^ ^)^ Moreover, inflammation also contributes to VEGF up-regulation.^31^ Indeed, cytokines such as interleukin (IL)-1 and IL-6, which are present at high levels during acute Chagas cardiomyopathy,^32^ also induce VEGF production.^31^

We verified higher levels of this transcription factor in parallel with parasitemia peak, suggesting the occurrence of hypoxia in the cardiac tissue when a high number of parasites is observed in blood and few inflammatory cells are present in cardiac tissue. Higher levels of HIF-1α positive cells were also observed in the cardiac sample from patients with chronic Chagas cardiomyopathy.^33^ At 15 dpi, when we observed vascular growth and peak of inflammation, the levels of HIF-1α were reduced in comparison to the levels at 8 dpi, when the quantity of inflammatory infiltrate is similar to noninfected controls, suggesting that inflammation is not the inducer of HIF-1α in acute CD.

We observed that, when compared to noninfected controls, a VEGF level was significantly higher, when the peak of inflammatory infiltrate and cardiac parasitism occurred (at 15 dpi). It has been reported that tissue over-expression of VEGF disturbs angiogenesis and induces vascular hyperpermeability,^34^ which could contribute to the entrance of inflammatory cells in the cardiac tissue. Cardiac muscle cells are the major source of VEGF in the heart and this protein can be up-regulated in injury situations.^35^ A systematic review was conducted and the meta-analysis also demonstrated that important VEGF levels were higher in patients with glioma, which play a crucial role in angiogenesis.^36^ Another study described that VEGF levels were significantly higher in the severe group of coronavirus disease 2019 (COVID-19). Kong et al. ^(^ ^37^ also suggested VEGF as a potential biomarker for detecting the progression of COVID-19.

Here, we also investigated the expression of Flk-1 and pERK. Flk-1 responds to VEGF stimulation through the activation of multiple signalling pathways involved in the regulation of essential steps for proper angiogenesis.^38^ Such steps include an increase in cellular migration, survival and cellular proliferation, vasodilatation and vascular permeability. Flk-1 expression increases concomitantly with VEGF-A increase and parallels an active angiogenesis process.^39^ Western blotting revealed an unexpected reduction on Flk-1 expression followed by a striking increase in this protein. Not only endothelial cells express Flk-1, but also inflammatory cells, specifically. T cells exhibit VEGF receptor 2 and also are capable of producing VEGF that acts autocrinally. VEGF acts in T cells inducing interferon (IFN)-γ and inhibiting IL-10 secretion.^40^

ERK1/2 activation is also involved in endothelial cell proliferation.^11^ ^,^ ^41^ Immunoblot analysis revealed induction of ERK1/2 activation when compared to noninfected controls, concomitant with the VEGF-A and Flk-1 higher levels. These results are in accordance with previous studies by our group showing increased levels of pERK in heart of CD-1 mice infected with the Brazil *T. cruzi* strain when compared to noninfected controls, concomitant with the reduction on caveolin-3 expression.^42^

Since our data demonstrated alteration in molecular mechanisms involved in angiogenesis in acutely *T. cruzi*-infected animals, we further examined the number of blood vessels in cardiac tissue of noninfected and *T. cruzi*-infected mice. Our results showed transient exacerbated angiogenesis in the heart tissue of *T. cruzi*-infected mice. Additionally, immunohistochemical analysis revealed an increase in CD31 staining at 15 dpi in the *T. cruzi* infected cardiac tissue, when compared to noninfected mice, supporting the occurrence of angiogenesis at this time point. CD31 can also stain inflammatory and endothelial progenitor cells.^43^ Further, this molecule is also involved in cellular migration and platelet aggregation.^44^

During the vascular maturation process, the α-SMA+ cells - pericytes and vascular smooth muscle cells - adhere to the abluminal vascular surface, providing stability to the newly formed blood vessel.^20^ By immunohistochemistry, we showed an increase in the number of α-SMA+ blood vessels in the cardiac tissue of *T. cruzi*-infected mice, when compared to noninfected mice. However, at 22 dpi, when the number of blood vessels decreased, even when there were higher levels of VEGF, at this time, we observed α-SMA+ cells covering blood vessels, close to them, and scattering among cardiac fibres, suggesting a vascular origin of myofibroblasts in *T. cruzi* infected cardiac tissue. Although α-SMA work as a vessel stabiliser, in the present work this role was not observed. 

It is possible that other molecules involved in vascular stabilization, including PDGF, TGF-β and Ang-1, for example,^45^ could be changed at 22 dpi. Studies of fibrogenesis in the liver^46^ ^,^ ^47^ ^)^ and kidney^48^ suggest that mural cells can be a source of myofibroblast progenitors, strongly supporting the role of angiogenesis in fibrosis.^47^ ^,^ ^49^ Finally, we assessed the presence of fibrosis in the cardiac tissue of acutely infected mice. We observed collagen-positive staining in the perivascular region and infiltrating the cardiac tissue. Since, mural cells can acquire a myofibroblast phenotype and actively produce ECM proteins, including collagen, the angiogenesis process can contribute to tissue remodelling in chronic inflammatory disorders.^15^ Interestingly, an increase of α-SMA staining in cardiac tissue was also observed in a murine model of chronic CD.^50^

Patients treated with the antibody anti-TNFα infliximab presented a reduction in VEGF expression and improvement of the symptoms of psoriatic arthritis.^51^ Interestingly, in the experimental model of chronic CD in C57BL/6 mice infected by the Colombian *T. cruzi* strain, the inhibition of TNF by treatment with infliximab reversed the immunological unbalance, the cardiac electrical alterations, and did not reactivate cardiac parasitism.^24^ Additionally, in an experimental model of rheumatoid arthritis in mice, the treatment with antibody anti-VEGFR-1 reduced synovial neovascularization and inflammatory infiltrates.^52^ In this context, we can speculate that the protective effect of infliximab during the CD^24^ ^)^ can also be associated with VEGF modulation.

How much angiogenesis is beneficial or deleterious in Chagas cardiomyopathy still needs to be investigated. The observations in the current report demonstrate that acute CD alters molecules involved in the regulation of vascular growth and causes exacerbated and transient angiogenesis. Transient angiogenesis is observed in inflammatory diseases contributing to tissue fibrosis through the α-SMA positive cells detachment from the vascular wall also resulting in vessel destabilization.^15^ ^,^ ^46^ ^-^ ^49^ In this study, the presence of α-SMA+ cells (myofibroblasts) near blood vessels in *T. cruzi* infected animals suggests a vascular origin of these cells. Thus, given our findings, we should consider the possibility that angiogenesis contributes to the genesis of cardiac fibrosis in acute *T. cruzi* infection, and future studies need to be addressed to confirm this issue.

In conclusion, our data demonstrated that *T. cruzi* acute infection in mice induces exacerbated angiogenesis, which can contribute to cardiac remodelling in Chagas cardiomyopathy. Thus, we strongly believe that in the future the angiogenic signalling pathway may be a promising chemotherapeutic target in CD.
